# Membrane resonance of bursting neuron captured with an I_Ca_/I_h _model using multi-objective evolutionary algorithms

**DOI:** 10.1186/1471-2202-14-S1-P289

**Published:** 2013-07-08

**Authors:** David Fox, Hua-an Tseng, Horacio G Rotstein, Farzan Nadim

**Affiliations:** 1 Department of Biological Sciences, NJIT-Rutgers University, Newark, NJ 07102, USA; 2 Department of Mathematical Sciences, NJIT, Newark, NJ 07102, USA

## 

Membrane potential resonance, a peak in the membrane impedance amplitude (|*Z*|) in response to oscillatory input current at a preferred frequency, is a common property of many neurons. It arises from interactions between passive membrane properties and voltage-gated ionic currents and has been implicated in the production of subthreshold and network oscillations. The PD neurons, one of the two cell types in the pyloric pacemaker group of the crab pyloric CPG, shows membrane resonance with a preferred frequency (*f_max_*= ~1 Hz) which is correlated with the network cycle frequency. The presence of membrane resonance in the PD neurons is sensitive to blockers of calcium currents *I_Ca _*and the hyperpolarization-activated inward current *I_h_*.

We used a single-compartment biophysical model comprising *I_leak_*, a low-threshold inactivating *I_Ca _*and *I_h _*to capture the membrane impedance profile (*Z *vs. *f_input_*) and the resonance in PD neurons. The impedance profile of the biological PD neuron and the model neuron were measured using a logarithmic sweeping-frequency (0.1 to 4 Hz) sinusoidal input (*A *sin(2π*f*(*t*)*t*)) over 100 sec. The model parameters were constrained using biological data. We used the multi-objective evolutionary algorithm, non-dominated-sorting genetic algorithm (NSGA-II ) to optimize five measurements defining the impedance profile: *f_max_, Z_max_, Q *(resonance power), *f*_1/2 _(half-width) and *Z*_fmax _which were characterized into objective functions to be minimized in the optimization process. The MOEA consisted of a generational population of 416 and iterated for 250 generations. The output consisted of trade-off solutions that assigned more importance to one objective over others. The parameter sets in the final generation were further restricted to those which fit all five objectives to within 10% of the biological data to obtain a final optimal group of models, all of which would be considered a good fit to the experimental PD impedance profile. The dependence of the objectives on the parameters was explored by subjecting all 15 parameters for all 35 models in the optimal group to a local sensitivity analysis.

The distribution of the parameters, both in the optimal group and in the final generation, shows a tight constraint on the leak conductance as well as the half-activation (*V*_½_Ca_act_) and half-inactivation (*V*_½_Ca_inact_) of *I_Ca _*and the half-activation (*V*_½_h_act_) of *I_h _*but moderate constraints on *g_Ca _*and *g_h _*and little constraint on the activation/inactivation time constants. The parameter sensitivities show that, across all 35 models, positive changes in *V*_½_Ca_act _can increase *Q *and decrease *f*_1/2 _(more peaky resonance profile). There is also an antagonistic relationship between *V*_½_Ca_act _and *V*_½_Ca_inact_: the former decreases whereas the latter increases *f_max_*. A pairwise-correlation analysis of all parameters shows that the time constants and conductance strengths seem to co-vary in order to maintain the shape of the impedance profile.

Many neurons display emergent properties in response to oscillatory inputs, such as amplified responses in certain frequency bands. These properties may be important in shaping coherent network activity. The underlying nonlinearities and time scales that shape specific features of impedance profiles can be used to link sub-threshold dynamics to supra-threshold voltage responses. We use the results of the optimization of the impedance profile to predict the spike phase of a neuron in an oscillatory network (presented separately at this meeting). We also plan to use the results of the sensitivity analysis to formulate predictions about the influence of resonant profile features on ongoing pyloric network activity.

## References

[B1] Hutcheon B Yarom Y Resonance, oscillation and the intrinsic frequency preferences of neurons TINS 2000 23 5 216 222 10782127 10.1016/s0166-2236(00)01547-2

[B2] Tohidi V Nadim F Membrane resonance in bursting pacemaker neurons of an oscillatory network is correlated with network frequency J Neurosci 2009 29 20 6427 6435 10.1523/JNEUROSCI.0545-09.2009 19458214 PMC2716082

[B3] Deb K Pratap A Agarwal S Meyarivan T A fast and elitist multiobjective genetic algorithm: NSGA-II Ieee T Evolut Comput 2002 6 2 182 197 10.1109/4235.996017

